# Co-Encapsulation of Phycocyanin and Albumin-Bound Curcumin in Biopolymeric Hydrogels

**DOI:** 10.3390/ijms26083805

**Published:** 2025-04-17

**Authors:** Konstantina Matskou, Ilias Matis, Sotiria Demisli, Konstantinos Rigkos, Eirini Karandrea, Kalliopi Kourioti, Georgios Sotiroudis, Vasiliki Pletsa, Aristotelis Xenakis, Maria Zoumpanioti

**Affiliations:** 1 Institute of Chemical Biology, National Hellenic Research Foundation, 11635 Athens, Greece; kmatskou@eie.gr (K.M.); iliasmatis@eie.gr (I.M.); sdemisli@eie.gr (S.D.); krigos@eie.gr (K.R.); karan.eirini@gmail.com (E.K.); kallikour95@gmail.com (K.K.); gsotir@eie.gr (G.S.); vpletsa@eie.gr (V.P.); arisx@eie.gr (A.X.); 2 Department of Biology, National and Kapodistrian University of Athens, 15701 Athens, Greece; 3 Department of Biological Applications and Technology, University of Ioannina, 45110 Ioannina, Greece

**Keywords:** antioxidants, biopolymers, hydrogels, structure, morphology, cell viability

## Abstract

Co-encapsulation of hydrophilic and hydrophobic compounds within a single delivery system remains a significant challenge across various scientific and industrial fields. Towards this direction, an encapsulation strategy is proposed, enabling the simultaneous incorporation of both hydrophilic and hydrophobic biomolecules within a hydrogel matrix. Specifically, the cyanobacterial protein phycocyanin (hydrophilic), extracted and purified by dry *Arthrospira maxima* biomass, and curcumin (hydrophobic) bound to bovine serum albumin (BSA) were utilized. This approach facilitates the indirect entrapment of hydrophobic molecules within the hydrophilic hydrogel network. The structural and physicochemical properties of the resulting hydrogels were characterized using optical analysis, scanning electron microscopy (SEM), and confocal laser scanning microscopy (CLSM). Additionally, the antioxidant potential of the encapsulated biomolecules was evaluated to assess their functionality after the encapsulation. Furthermore, a cell viability assay confirmed the hydrogel’s biocompatibility and lack of toxicity, demonstrating its suitability as a multifunctional biomaterial for biomedical and pharmaceutical applications.

## 1. Introduction

Hydrogels have gained increasing attention due to their versatile applications in formulation science, particularly for encapsulating nano- and macro-molecules, through various interactions or entrapment within their three-dimensional polymer networks [1,2]. For biological applications, hydrogels require biocompatible and biodegradable polymers, with natural options like chitosan and cellulose playing a crucial role.

Chitosan, derived from chitin via deacetylation, is valued for its biodegradability, antimicrobial properties, and cationic nature, enabling interactions with various polysaccharides while offering mechanical flexibility [3]. Similarly, (hydroxypropyl)methyl cellulose (HPMC) is a water-soluble polymer widely used for its biocompatibility, biodegradability, swelling capacity, and hydrophilicity. These properties make chitosan–HPMC hydrogels promising candidates for biomedical and pharmaceutical applications [4,5]. Overall, hydrogels are a common choice for encapsulating and delivering hydrophilic compounds, as these are easily dispersed within the matrix of hydrogels.

While hydrogels are widely used for the encapsulation of hydrophilic compounds, the incorporation of hydrophobic bioactive molecules into these matrices remains a significant challenge. Current strategies, including polymer modifications and surfactant-assisted systems, seem promising but require further optimization to enhance encapsulation efficiency and stability [6,7]. Additionally, research on simultaneous encapsulation of both hydrophilic and hydrophobic compounds remains limited, despite its potential to unlock synergistic effects in drug delivery and other fields [8].

Various hybrid methods have been explored to address the incorporation of hydrophobic compounds into hydrophilic environments, e.g., the use of surfactant-stabilized microemulsion droplets, surfactant micelles, and polymer micelles [9]. Nevertheless, the incorporation of secondary carriers in these approaches often results in pronounced aggregation during the loading process. This phenomenon contributes to an exceedingly heterogeneous dispersion of the drug within the hydrogel, significantly impacting the formulation’s usability [10].

A prior investigation by our group sets the foundation for a breakthrough in this impasse by employing a soluble protein carrier i.e., bovine serum albumin (BSA) [11]. The resulting protocol facilitates the binding of BSA by a lipophilic compound, namely, curcumin, to bind to the protein BSA, allowing it to homogeneously disperse within the aqueous solution.

Curcumin (CCM) is characterized by an extensive array of pharmacological effects, such as its ability to combat tumors, its antioxidant activity, an ability to counteract amyloid formation, and its anti-inflammation properties [12]. Its ability to bind albumin is well characterized. It gets located in the vicinity of the two Trp-containing hydrophobic pockets, effectively counteracting its inability to solubilize in water under physiological conditions [11].

Phycocyanin is a light-harvesting, hydrophilic protein produced in cyanobacteria, such as those of the genus *Arthrospira*. It is a phycobiliprotein, i.e., a family of proteins covalently attached to open chain tetrapyrroles known as phycobilins. Phycocyanin has seen widespread use in the food and cosmetics industry owing to its natural blue color. It has also been studied in research applications as a fluorescent marker and in pharmaceutical applications for its antioxidant and anti-inflammatory properties [13].

The study presented here tests our proposed gel formulation approach by encapsulating a hydrophilic bioactive protein, namely, phycocyanin, along with a hydrophobic polyphenol in the form of BSA-bound curcumin, in chitosan and/or HPMC polymeric hydrogels. These systems were studied morphologically and structurally, revealing their three-dimensional network. Since both phycocyanin and curcumin have antioxidant activities, the proposed loaded hydrogels were evaluated for their antioxidant ability, revealing overall positive effects which were more pronounced for BSA-bound curcumin. The cell viability assay revealed no cytotoxicity for either empty or antioxidant-loaded hydrogels, while the antioxidant activity of the encapsulated compounds was maintained after encapsulation.

## 2. Results

### 2.1. Phycocyanin Extraction and Purification

Phycocyanin extraction was achieved following the protocol described in Section 4.2.1. After extraction, the absorbance spectrum at 200–700 nm was used to calculate the final phycocyanin concentration, purity, and extraction yield. The highest phycocyanin concentration that was retrieved was 6.18 mg/mL and the extract purity (EP) was 2.74, similar to that reported in the literature [14,15]. This value is important regarding downstream applications, as a value between 0.7 and 4 is required for the food industry [16]. The yield was 60.2 mg/g of biomass. The high purity degree of the isolated phycocyanin was also indicated by the bright blue color of the isolate (Supplementary Material Figure S1). As can be seen in Figure S1, the color is bright and with no visible contamination. The most common contaminants at this stage are related to the presence of chlorophyll that provides a characteristic green color [15].

To confirm the results of the absorbance measurements, 12% SDS PAGE was carried out (the results are presented in Figure S2). The gradual purification was observed, with the highest concentration of the desired protein being detached when 150 mM PB was added to the column. The usage of 200 mM PB also facilitated the release of phycocyanin, but 250 mM PB did not show efficient results, meaning that the optimum PB concentration for phycocyanin release is 150–200 mM. Interestingly, blue color remained on the column after increasing the PB concentration. For the column cleaning process, 1 M NaCl solution was used and most of the blue color was removed from the column. A sample of this occurring solution was used in SDS PAGE, and it was clear that, although it contained an important amount of phycocyanin, the sample was contaminated with other proteins that were also attached to the column.

### 2.2. Hydrogel Characterization

#### 2.2.1. Heating Treatment

Several hydrogels were prepared aiming at the encapsulation of the isolated phycocyanin as well as CCM/BSA. The hydrogels were then characterized in terms of thermal stability as their application in various fields, such as their use as stabilizing agents of biotherapeutic drugs, depends on their tolerance of temperature changes [17].

**Figure 1:**
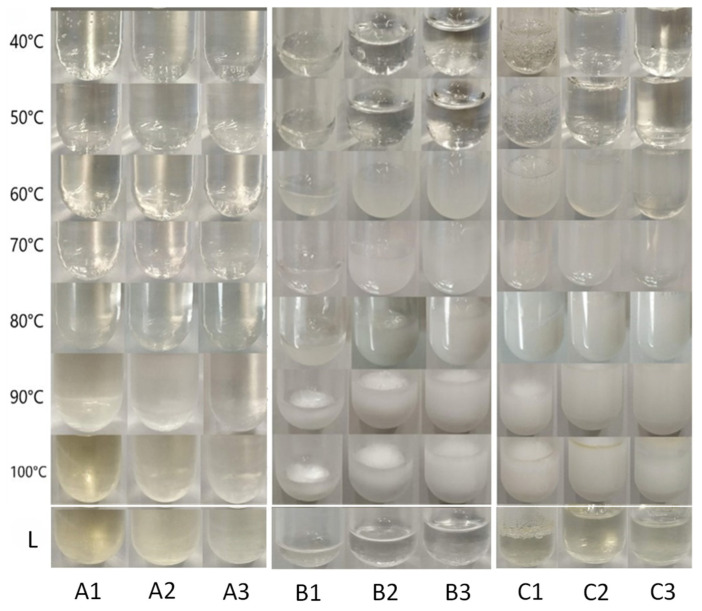
Hydrogels after incubation at different temperatures for 1 h. From left to right: systems based on chitosan (A1, A2, A3), HPMC (B1, B2, B3) and hybrid chitosan/HPMC hydrogels (C1, C2, C3). A1–C3: refer to Table 1. L: the systems 3 d after the heating study.

The thermal stability study was carried out for systems presented on Table 1. The hydrogels were incubated in a heated waterbath for 1 h at each set temperature (40 °C to 100 °C). Photographs were taken at each step as well as three days after the heat treatment procedure for a better comparison. After the study was completed, the photographs were used to evaluate changes in characteristics such as color, structure, and clarity. The fluidity of the systems was also checked throughout the study. The results for each individual type of hydrogel are presented in Figure 1.

##### Chitosan Hydrogels

Observing the chitosan-based hydrogels (Table 1, systems A1, A2, and A3), no particular change of the systems was detected responding to the different temperatures applied, up to 80 °C. The results of the study for these systems are presented in Figure 1 (left). A noteworthy observation is that after incubation at 90 °C the systems became slightly yellow and turned even more so at 100 °C. Our results showed that Systems A1 and A2 in which chitosan content was higher, developed a more intense yellow color. Furthermore, the color did not revert after three days; however, all systems were stable regarding visible observation and no change in their relative fluidity was observed. The yellow color observed after 90 °C can be attributed to the degradation of chitosan at elevated temperatures [18,19]. According to the literature, chitosan undergoes thermal degradation in two stages, with the first significant mass loss occurring around 90 °C. At this temperature, approximately 5.8% mass loss is observed, indicating that chitosan begins to degrade under such conditions. This phenomenon is not reversible when the temperature returns to normal conditions, as the polymer undergoes chemical and physical changes that can lead to the breakdown of its polymer chains. This process typically results in the formation of smaller molecular weight fragments and loss of functional groups [18,19].

##### HPMC Hydrogels

(Hydroxypropyl)methyl cellulose (HPMC) exhibits notable thermal stability, which is essential for various applications, particularly in pharmaceuticals and materials science. When the HPMC-based systems (Table 1, systems B1, B2 and B3) were tested against increased temperature, they maintained their clarity up to 50 °C. Then, at 60 °C and 70 °C, the systems acquired a white color alongside increased turbidity, while at higher temperatures, the white color became even more intense as shown in Figure 1 (middle). This phenomenon was reversible as three days later the systems regained the clarity and color they had before heating.

HPMC solutions exhibit changes in turbidity as they approach their gelation temperature. Between 60 °C and 70 °C, an HPMC solution can become increasingly turbid due to the aggregation of polymer chains and the onset of gelation processes. At these temperatures, the hydrophobic domains of HPMC start to interact more significantly, leading to aggregation and cloudiness in the solution [20]. Upon cooling, these interactions can reverse, allowing the hydrogel to return to a more soluble state, which typically results in decreased turbidity [20].

##### Chitosan–HPMC Hydrogels

To enhance thermal stability, researchers often explore blending chitosan with other polymers or incorporating stabilizers that can mitigate degradation at higher temperatures. Such a mixture often involves HPMC.

The systems based on the chitosan–HPMC mixture (Table 1, systems C1, C2 and C3) showed similar behavior to the ones based on HPMC. Clarity was maintained up to 50 °C, while at 60 °C and 70 °C, a strong white color appeared which then became even stronger at higher temperatures (Figure 1, right). Interestingly, as happened to the chitosan systems, at temperatures of 90 °C and 100 °C, a slight yellow color co-exists with the white color. Three days after the heating study (Line L in Figure 1), the bright white color disappeared completely and the yellow color prevailed. This is in accordance with the results presented here for chitosan-based and for HPMC-based hydrogels. In the case of chitosan/HPMC blends, turbidity is reversible when the temperature reverts to normal conditions, but the coloring attributed to chitosan degradation at 90–100 °C is irreversible.

The fluidity of all the samples (A1 to C3) did not change during the study.

#### 2.2.2. Swelling Ratio

The swelling ratio provides insights into the ability of a hydrogel to absorb water or any aqueous solution, which is crucial for applications in drug delivery, tissue engineering and other biomedical fields. To draw conclusions on the ability of the hydrogels to absorb any aqueous solution, the hydrogels were initially dried using the freeze-drying method. Then, the dried hydrogels were dipped in phosphate-buffered saline for 15 min to allow rehydration. After that time, their weight was monitored in order to calculate the weight gain and, consequently, the water absorption. A time of 15 min was chosen as further hydration led to partially dissolved hydrogels. The study was performed with empty as well as PC-loaded hydrogels.

**Figure 2:**
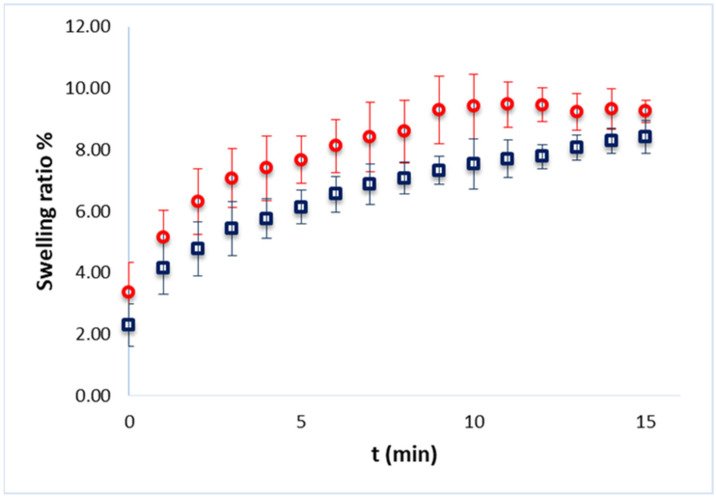
Percent swelling ratio of empty (red) and PC-loaded (blue) hydrogel (system C1 of Table 1), as a function of the incubation time.

Figure 2 presents the percent swelling ratio as calculated using Equation (4) for the system C1 of Table 1, empty or loaded with PC. As can be seen, the empty hydrogel showed a slightly higher swelling capacity compared to the PC-loaded hydrogel. This can be attributed to the presence of PC in the network. The swelling behavior of hydrogels is influenced by their network structure and the interactions between polymer chains and water. The study by Ramli et al. [21] highlighted that a more porous network allows for greater water absorption, while the addition of other components can increase the density of the network, thereby reducing swelling capacity. This aligns with our observation that PC increases the density of the hydrogel matrix, making it more difficult for water to penetrate. Also, the interactions between phycocyanin and HPMC can enhance the mechanical properties of the hydrogel but also contribute to a denser structure that limits water absorption. This has been observed in various formulations where the presence of certain additives alters both mechanical and swelling characteristics [22].

#### 2.2.3. Morphological Analysis

**Figure 3:**
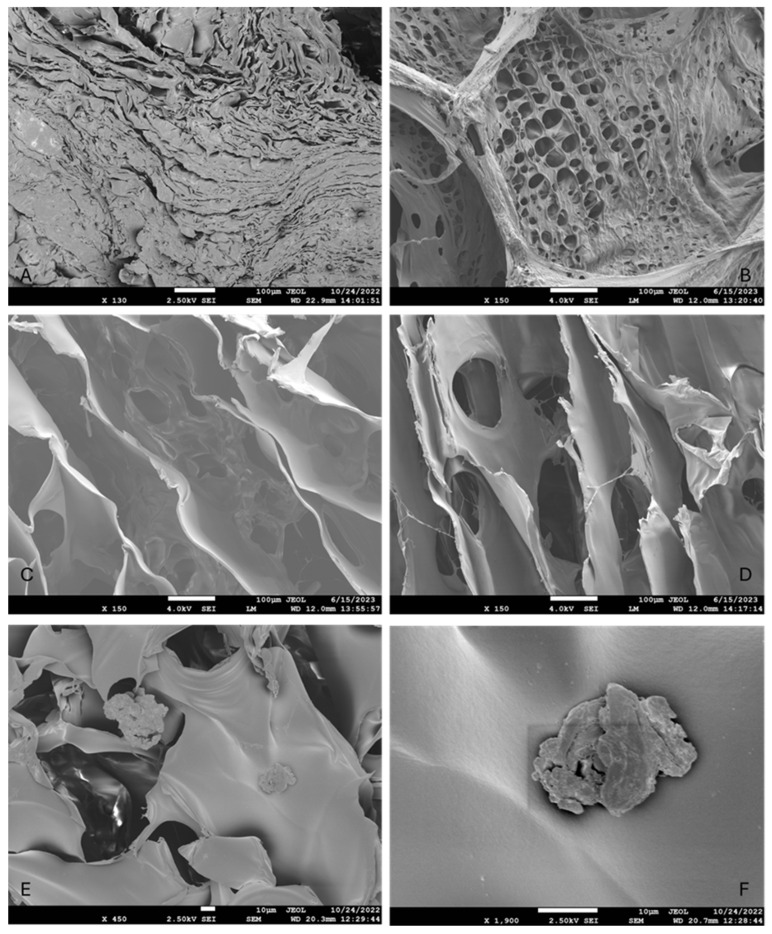
SEM images of the freeze-dried hydrogels. (**A**): empty hydrogel; (**B**): PC-loaded hydrogel; (**C**): CCM/BSA-loaded hydrogel; (**D**): CCM/BSA and PC-loaded hydrogel; (**E**): magnified area of the CCM/BSA-loaded hydrogel where aggregates are visible; (**F**): zoomed-in aggregate in CCM/BSA-loaded hydrogel.

The morphology of the empty as well as antioxidant-loaded hydrogels was observed via scanning electron microscopy (SEM) images that revealed a three-dimensional network. In Figure 3, the images taken for freeze-dried hydrogels are presented. As can be seen, the empty hydrogel (Figure 3A) appeared to be more compact, with more condensed folds and fewer pores. When any antioxidant was added (Figure 3B–D), the hydrogel appeared to develop loose and highly porous folds and filaments. In the case that CCM/BSA was added, either alone (Figure 3C) or combined with PC (Figure 3D), the pores appeared to be larger than the ones developed when PC was immobilized (Figure 3B). The more compact nature of the empty hydrogel has also been the result of a previous study concerning a similar chitosan–HPMC-based film [23]. According to this study [23], the empty chitosan/HPMC system was smooth and without pores. Other studies also confirm the non-porous nature of empty HPMC [24] or gelatin [25] systems that develop pores after a component’s encapsulation.

The differences in porosity observed in the hydrogels can be explained by a combination of polymer–protein interactions, molecular crowding, and crosslinking effects. Chitosan and the encapsulated proteins (phycocyanin, BSA) carry different charges depending on the pH, leading to electrostatic interactions that influence the polymer network structure [26]. Additionally, both chitosan and HPMC contain hydroxyl and amine groups that can form hydrogen bonds with proteins, which may affect the gel’s overall integrity. Proteins can also act as physical crosslinkers, altering network density and leading to variations in porosity depending on their affinity and spatial arrangement within the gel.

The water retention capacity of the hydrogel is another key factor affecting porosity. Some proteins, such as BSA, can bind water molecules, impacting hydrogel swelling and preventing tight polymer interactions, which may lead to a more open and porous structure. Phycocyanin, due to its globular structure and high water-binding capacity, may contribute to a more hydrated and porous gel matrix. On the other hand, BSA, especially when loaded with curcumin, can act as a spacer within the hydrogel, disrupting polymer packing and influencing hydrophobic interactions, which may further impact porosity. When both proteins are present, competition for binding sites or phase separation effects could lead to a distinct porous structure compared to hydrogels containing only one protein.

Additionally, the gelation rate plays a role in defining the hydrogel’s final structure. Proteins can influence how polymer chains organize during gelation, potentially creating microdomains if they do not fully integrate within the matrix. This may contribute to heterogeneous porosity, particularly when multiple proteins are involved [27]. Overall, the variations in hydrogel porosity can be linked to a balance of electrostatic interactions, hydration effects, and polymer–protein compatibility, which determines the final network architecture.

From the images taken in our study, it was also noticed that in the case of the sole addition of CCM/BSA, scattered aggregates could be observed, which are believed to be protein aggregates that resulted from the freeze-drying process and the rapid dehydration of the system (Figure 3E,F).

#### 2.2.4. Structural Characterization

**Figure 4:**
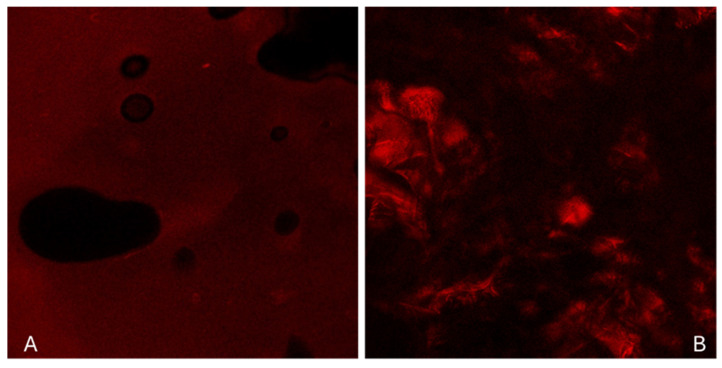
(**A**): Confocal image ×20 magnification of the surface of hydrogel C4 loaded with phycoerythrin and (**B**): Confocal image ×20 magnification of a cross-section of the same system. Composition of hydrogel C4 as given in Table 1.

For the structural characterization of hydrogels, confocal scanning electron microscopy (CLSM) was applied. The results for the hydrogel C4 (system C4 of Table 1) are shown in Figure 4. As can be seen from the left image (Figure 4A) that depicts the surface of the hydrogel, its porous nature is verified. In the image of the cross-section (Figure 4B), the chaotic network resulting from the chemical interaction of the two polymers can be observed. The varying intensities of red fluorescence, indicating the presence of phycoerythrin, suggest that it has diffused unevenly. Specifically, phycoerythrin probably tends to concentrate in areas of the hydrogel dominated by one of the two polymers. Chitosan as a cationic polymer contains amino groups (-NH2) that can form strong hydrogen bonds and electrostatic interactions with the negatively charged regions of phycoerythrin. This enhances the stability and solubility of phycoerythrin within the hydrogel matrix. It is also known that chitosan remains soluble in acidic conditions but precipitates and forms a gel as the pH increases above approximately 6.5, a pH value beneficial for phycoerythrin stability [28].

### 2.3. Antioxidant Ability Study

The antioxidant activity of two antioxidant agents, namely, PC and CCM/BSA, free in aqueous solution or incorporated in hydrogels, was studied and the results are summarized in Table 2. The antioxidant activity was expressed as a percent reduction of DPPH. In every case, the standard deviation was less than 0.1 (Table 2).

As can be seen from Table 2, the empty hydrogel appears to have antioxidant activity. This ability can be attributed to the presence of chitosan in the hydrogel, which acts as an antioxidant agent [29].

When the studied antioxidants are solubilized in aqueous solution, CCM/BSA appears to have a slightly higher activity than PC. The antioxidant activity increases when both antioxidants are present. Similar behavior is evident when each of the antioxidants is incorporated into the hydrogel, where the CCM/BSA-loaded hydrogel has a higher activity than the PC-loaded hydrogel, although in this case, the activity is not higher when both antioxidants are present.

Comparing the activity of dispersed in water antioxidants to their activity when incorporated in the hydrogels, we can observe that the activity of incorporated PC appears to be restricted. It appears that encapsulation can restrict its mobility and availability to interact with free radicals, thereby diminishing its antioxidant effectiveness. A reason for this observation could be the fact that the stability of phycocyanin can be affected by the hydrogel environment. Factors such as pH, temperature, and ionic strength can influence its structural integrity [30]. On the contrary, from Table 2, it can also be seen that in the case of incorporated CCM/BSA as well as in the case that both antioxidants are incorporated, the antioxidant activity is enhanced when compared to the one presented by free antioxidants in an aqueous solution. This can be attributed to the presence of chitosan which may act synergistically. Also, enhancement in antioxidant capacity has been observed when chitosan is chemically modified, such as through the incorporation of polyphenols or other antioxidants that synergistically improve its free radical scavenging ability [31].

### 2.4. Cell Viability Assay

To verify the biocompatibility of the proposed hydrogel, a cell viability assay was conducted by MTT. This assessment provides essential insights into the safety and effectiveness of the materials used. The inhibition of cell proliferation by empty as well as loaded (PC, CCM/BSA, PC/CCM/BSA) systems was tested in WM164 melanoma cells, following the spreading and crosslinking of the hydrogel at the bottom of each well in 96-well plates to form a matrix on which cells were settled to grow [32,33].

**Figure 5:**
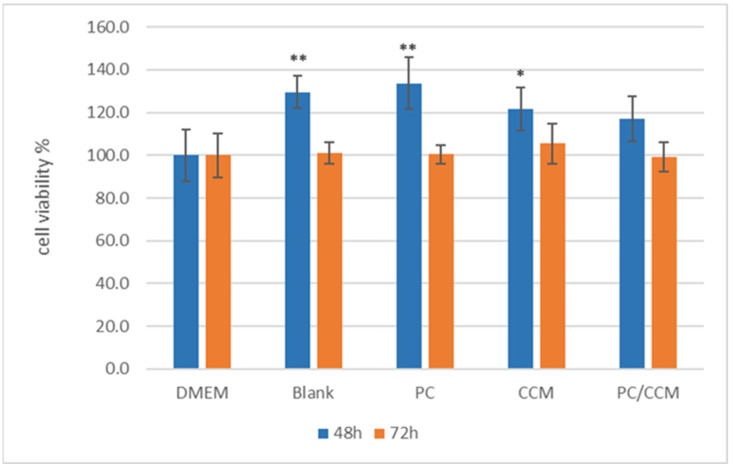
Thiazolyl blue tetrazolium bromide (MTT) cell proliferation assay with the WM164 cell line after 48 and 72 h in the presence of 10 µL crosslinked hydrogel spread at the bottom of the wells as a basis for cell growth: Dulbecco’s Modified Eagle’s Medium (DMEM); empty hydrogel, 10.4 mg (blank); hydrogel loaded with PC (PC), 10.4 mg/4 µg; hydrogel loaded with CCM/BSA (CCM), 10.4 mg/0.02 µg; hydrogel loaded with both PC and CCM/BSA (PC/CCM), 10.4 mg/4 µg/0.02 µg. The mean (±SD) of three independent experiments, each performed in five replicates, is presented. Statistically significant results are indicated by asterisks (*) when *p* < 0.05 and (**) when *p* < 0.01 (One-Way ANOVA with Bonferroni correction for multiple testing).

No inhibition of cell proliferation was observed (Figure 5) when cells were grown in the presence of 10 µL of the hydrogels for 48 and 72 h. On the contrary, at 48 h, an enhancement in cell proliferation was observed in the presence of the hydrogel. This transient enhancement could be expected when hydrogels, acting as a network facilitating cell migration and communication [32], are combined with cell culture conditions favoring exponential growth of WM164 cells, which is the case during the first 48 h of treatment. At 72 h, the viability in the presence of the empty as well as the loaded hydrogels was similar to that of the control untreated cells (DMEM) (Figure 5).

## 3. Discussion

During this study, several hydrogels based on chitosan or/and HPMC were prepared (Table 1) with the aim of using them for antioxidants encapsulation. The thermal stability assessment of the systems demonstrated that chitosan-based hydrogels remained structurally stable but developed irreversible yellowing at temperatures of 90 °C and above, a result consistent with the reported thermal degradation of chitosan [18]. In contrast, hydrogels containing (hydroxypropyl)methyl cellulose (HPMC) exhibited reversible turbidity at 60–70 °C, which cleared upon cooling. The chitosan–HPMC hybrid hydrogels combined the properties of their individual components, maintaining clarity up to 50 °C, showing temporary turbidity at 60–70 °C, and exhibiting permanent yellowing at 90–100 °C. Despite these visible changes, fluidity remained unaffected across all formulations, indicating that thermal exposure did not significantly alter the rheological properties of the hydrogels.

Swelling ratio analysis confirmed the high-water absorption capacity of the hydrogels. However, the incorporation of phycocyanin led to a slight decrease in swelling capacity, likely due to increased density restricting water uptake [21]. The addition of HPMC can enhance the mechanical properties of the hydrogel but also contributes to a denser structure that limits water absorption [22]. Variations in phycocyanin concentration did not significantly impact the overall swelling behavior, suggesting that once incorporated, phycocyanin does not significantly affect the hydrogel’s ability to retain water.

Morphological analysis using scanning electron microscopy (SEM) revealed the formation of a three-dimensional porous network in the antioxidant-loaded hydrogels. The empty hydrogel appeared more compact, whereas antioxidant-loaded systems exhibited a more porous structure with loosely arranged folds and filaments. Moreover, hydrogels containing CCM/BSA displayed larger pores and some scattered protein aggregates, likely resulting from the freeze-drying process. Confocal scanning laser microscopy (CLSM) imaging of the hydrogel confirmed its porous architecture and revealed uneven diffusion of phycoerythrin, likely influenced by electrostatic interactions with chitosan and the pH-dependent gelation properties of the polymer.

Antioxidant activity analysis demonstrated that chitosan-containing hydrogels inherently exhibited antioxidant properties, likely enhanced by the free radical scavenging ability of chitosan. When tested in aqueous solution, CCM/BSA exhibited slightly higher antioxidant activity than phycocyanin. The combination of both antioxidants in water resulted in enhanced activity, a trend that was not so pronounced when they were incorporated into hydrogels. However, phycocyanin appeared to have a restricted mobility and limited antioxidant potential upon encapsulation. In contrast, the antioxidant activity of CCM/BSA was enhanced upon encapsulation, supporting a possible synergistic interaction with chitosan. The results further indicate that chitosan may contribute to the overall antioxidant activity, especially when chemically modified or combined with other bioactive compounds.

To evaluate the biocompatibility of the hydrogels, a cell viability assay was conducted by spreading and crosslinking 10.4 mg of the hydrogels as a scaffold for cell growth in 100 μL growth medium [33,34]. None of the hydrogel formulations exhibited cytotoxicity. On the contrary, cell viability was enhanced at 48 h, which is the doubling time for the WM164 melanoma cell line. In agreement with previous evidence [35], the presence of the hydrogel matrix, mimicking extracellular matrix, appeared to enhance cell proliferation due to its porous network which facilitates cell migration and intercellular communication. These results encourage further investigation and development of these hydrogels to be used as scaffolds in tissue engineering.

## 4. Materials and Methods

### 4.1. Materials

The *Spirulina maxima* dried biomass was kindly donated by the company Algh A.C., Greece. *Porphyridium purpureum* (formerly known as *P. cruentum*) powder was purchased from Alganex GmbH, Berlin, Germany. Di-sodium hydrogen phosphate, sodium dihydrogen phosphate (for phosphate buffers), ammonium sulfate, dialysis tubing (MWCO 12,000–14,000), and DEAE sepharose fast flow were obtained from Sigma, Darmstadt, Germany. Vivaspin^®^ Ultrafiltration falcons were obtained from Sartorius AG, Göttingen, Germany.

(Hydroxypropyl)methyl cellulose (HPMC) (3600–5500 cP) was obtained from Sigma, Darmstadt, Germany, and the chitosan (CS) from shrimp and other crustacean shells (viscosity 200–600 mPa·s, 0.5% in 0.5% Acetic acid, 20 °C; Deacetylation value: 80%, molecular mass 1526.464 g/mol) was purchased from TCI, Zwijndrecht, Belgium.

Fatty-acid-free bovine serum albumin (BSA) Fraction V (>98%) was purchased from PAN Biotech, Aidenbach, Germany. Curcumin (CCM) (>98%) was purchased from Apollo Scientific, Stockport, UK.

DPPH (2,2-diphenyl-1-picryl-hydrazyl-hydrate) and MTT assay (M5655) were obtained from Sigma, Darmstadt, Germany, and broad range prestained protein marker was obtained from Thermo-Fisher Scientific, Waltham, MA, USA. Coomassie brilliant blue R250 was obtained from Applichem, Darmstadt, Germany.

All aqueous solutions were prepared with ultrapure water (Siemens Ultra-Clear TWF system, Munich, Germany).

The human melanoma cell line WM 164 (BRAFV600E, p53Y220C; Wistar Institute Melanoma Research Centre, Philadelphia, PA, USA, https://wistar.org/) was kindly donated from the American Type Culture Collection, ATCC, Manassas, VA, USA.

### 4.2. Methods

#### 4.2.1. Phycocyanin Extraction and Purification

To obtain phycocyanin from *Spirulina maxima* biomass, an extraction and isolation procedure was followed. In this context, 0.1 M phosphate buffer (PB) (pH 7.0) was mixed with spirulina dried biomass (10% *w*/*v*) and left stirring overnight at 4 °C (dark, ambient temperature). After this step, any water-soluble ingredients were released from the dried biomass in the phosphate buffer. Then, the suspension was centrifuged for 10 min at 16,000× *g* (10 °C) and the supernatant was collected. The next step was the gradual protein precipitation to avoid other proteins extracted from the spirulina biomass being present in the final phycocyanin solution. Solid ammonium sulfate (AS) was added to the collected supernatant at 30% (*w*/*v*) saturation and the mixture was continuously stirred for 2 h at 4 °C. The solution was again centrifuged for 20 min at 16,000× *g* (10 °C) and AS up to 70% (*w*/*v*) was added to the supernatant under continuous stirring for 2 h at 4 °C. The solution was centrifuged again, and the precipitate was dissolved to a minimum volume of 50 mM PB pH 7.0. The following step was dialysis against the same buffer (for 2 days, changing the buffer daily) to expel the ammonium sulfate salts and any small molecules from the protein solution. After the dialysis procedure, a DEAE sepharose fast flow column was used for further purification. A gradient addition of 50 to 250 mM phosphate buffer (pH 7.0) was applied after the protein solution was inserted into the column and the protein was collected. Flow was kept at 40 mL/h. After the purification, solvent exchange was performed with repeated cycles of centrifugation in special ultrafiltration tubes of 50 kDa MWCO to exchange the phosphate buffer with distilled water.

Concentration and purity of the phycocyanin samples were determined by UV–visible absorption spectra at 620 nm, 650 nm (where allophycocyanin absorbs), and 280 nm [36]. Phycocyanin concentration (PC; mg/mL) was defined by the following Equation (1):

PC (mg/mL) = [OD_620_ − 0.474 OD_650_]/5.34 (1)

Purity of phycocyanin (extract purity, EP) was defined as the ratio of absorbance at 620 and 280 nm using the following Equation (2):

EP = OD_620_/OD_280_ (2)

The yield of C-phycocyanin extraction (mg/g) was calculated using phycocyanin concentration (CPC; mg/mL), the volume of solvent used for the extraction (V; mL), and the weight of the dried biomass (W_B_; gr) defined as the following Equation (3):

Yield (mg/g) = (CPC × V)/W_B_ (3)

A 12% SDS PAGE was carried out to confirm the purity of phycocyanin. The bands were visualized by Coomassie blue staining. Molecular weight of the purified phycocyanin was determined by a broad range prestained protein marker.

#### 4.2.2. CCM/BSA Protein Carrier Formulation

Briefly, lyophilized BSA was dissolved in distilled water, to a concentration of 50 mg/mL. CCM was dissolved in dichloromethane to create stock solutions of different concentrations. Then, 1 mL of the aqueous BSA solution was mixed with 0.25 mL of a stock CCM solution. The occurring two-phase system was vortexed briefly and was sonicated for 5 min using a BIORUPTOR^®^ Standard sonicator (Diagenode, Denville, NJ, USA). To achieve the final CCM/BSA formulation, the mixture was then transferred into a spherical flask and the organic solvent was evaporated at 30 °C in a waterbath under vacuum, using a Buchi Rotavapor R110 (Flawil, Switzerland) rotary evaporator [11].

#### 4.2.3. Hydrogel Formulation and Encapsulation of Antioxidants

##### Hydrogel Preparation

Two stock solutions were initially prepared, a chitosan in 1% acetic acid aqueous solution and a solution of HPMC in water, both at several concentrations. The tested systems were formed by mixing the above solutions in different ratios ranging from chitosan–HPMC solution 0:100% *v*/*v* to 100:0% *v*/*v*. It should be noted that the chitosan solution is acidic due to the presence of acetic acid. The pH of this solution was 3.5. The pH of the HPMC solution was found to be 7.5. After mixing, the overall pH changes due to dilution of the acidic medium and possible interactions between chitosan, which contains protonated amino groups, and HPMC. Indeed, the pH of the mixture was found to be 5.

For chitosan dissolution, heat and continuous mechanical stirring were required (65 °C for 20 min) to ensure complete solubilization, while the HPMC solution was easily prepared by simple stirring. Once both polymers were fully dissolved, the respective solutions were mixed at different ratios and homogenized by gentle stirring with a spatula to minimize air entrapment. The stability of the produced hydrogels was tested with a vial-tilt test. The hydrogels were stable and maintained the shape of the vial even when the vial was reversed (Supplementary Material Figure S3). It should be noted here that the gelation was not achieved by the addition of a cross linker. Chitosan and HPMC both have hydroxyl groups that can form extensive hydrogen bonds which may contribute to a structured network. The gelation also depends on the polymer concentrations. The mixture of the two polymer solutions is highly viscous, and after stirring with a spatula for just a few seconds, the gel occurs.

##### Encapsulation of Antioxidants in Hydrogels

For the preparation of chitosan–HPMC hydrogels that contained antioxidants, the optimum system in terms of polymer weight ratio was selected, based on stability and encapsulation ability of the produced hydrogel. The selected antioxidant was solubilized in the aqueous HPMC solution, which was then mixed with the chitosan solution in the desired ratio. This procedure was necessary for the proteinic antioxidants, as they could be denatured upon immediate mixing with 1% acetic acid solution (chitosan solution) because of the pH change.

Briefly, for the preparation of a hydrogel with final polymer content 1.8% *w*/*v* HPMC and 1.8% *w*/*v* chitosan, 0.09 g of chitosan was added to 3 mL of 1% aqueous acetic acid solution. The chitosan solution was heated to 65 °C for 20 min and mechanically stirred. After full solubilization of the chitosan was achieved, the solution was left to cool to room temperature. Then, a solution of 0.09 g of HPMC dissolved in 2 mL of water containing the antioxidant agent was added to the chitosan solution, followed by manual stirring with a spatula to homogenize the polymer mixture.

For the encapsulation of PC, 100 µL of an aqueous PC solution with a concentration of 20 mg/mL was introduced to the HPMC solution, followed by manual stirring with a spatula. Therefore, the final concentration of PC in the hydrogel (5 mL) was 0.4 mg/mL. For the hydrogel containing curcumin, 133 μL of CCM/BSA solution with a CCM concentration of 88 μg/mL was introduced to the HPMC solution so the antioxidant’s final concentration in the hydrogel was 2.34 μg/mL. For the calculations, it has been taken into account that approximately 80% of the initial amount of curcumin used to form the carrier (CCM-BSA) is bound [11]. The concentrations of the encapsulated substances were selected after performing antioxidant activity tests in aqueous solutions and then adapted, respectively, to the hydrogels.

#### 4.2.4. Hydrogel Structural Characterization

The hydrogels presented in Table 1 were chosen to carry out stability studies and to choose the most appropriate polymer ratio.

##### Thermal Stability

To evaluate the thermal stability of the empty hydrogels, visual observation was carried out prior to and after incubation at different temperatures, followed by cooling down to ambient temperature. Specifically, during incubation, temperature increased by steps of 10 °C from 40 °C up to 100 °C. On each step, the temperature was kept constant for 1 h. Temperature control was achieved by a heated water bath and after each temperature change, the changes in the hydrogels’ relative fluidity were recorded.

##### Swelling Ratio

To study the ability of the hydrogels to absorb water, the swelling ratio technique was applied. For this purpose, hydrogels were freeze-dried overnight in a freeze-dryer (Christ, Alpha LSCbasic, Osterode am Harz, Germany). After freeze-drying, the dry specimens were weighed (W_dry_) and then immersed in phosphate-buffered saline (PBS) (0.05 M) for 15 min. The hydrogels were then wiped off to remove liquids that remained on the surface and weighed again (W_swollen_). For the weighing, a high precision balance was used. The swelling ratio (SR) is defined by the following equation:

SR = [(W_swollen_ − W_dry_) × 100]/W_dry_ (4)

##### Scanning Electron Microscopy (SEM)

The morphology of the specimens underwent examination through field emission scanning electron microscopy (FE-SEM), conducted using the JEOL JSM-7610F, Tokyo, Japan instrument [37]. The hydrogels were freeze-dried before analysis and placed on either carbon (C) or copper (Cu) surfaces and then sputtered with Pd/Au using a Quorum SC7620 sputter/coater (Quorum Technologies Ltd., Laughton, East Sussex, UK) to enhance visualization. Imaging was carried out at a maximum operating range of 5 kV, and the micrographs were taken at increasing magnifications.

For the SEM structural characterization, the hydrogel corresponding to the C4 system in Table 1 was used, either empty or loaded with PC, CCM/BSA, and both of them combined.

##### Structural Characterization

The microscopical analysis of the hydrogels was conducted by confocal scanning electron microscopy (CLSM). For structural characterization, researchers often use fluorescent probes to visualize and analyze the structure and properties of hydrogels. For this purpose, the hydrogel (C4 system in Table 1) was studied loaded with phycoerythrin at a concentration that matched that of phycocyanin. Phycoerythrin is a fluorescent protein. Its strong fluorescent response and the large difference between absorption and emission (Stokes shift) make it ideal for fluorescence applications [38]. Phycoerythrin was isolated in the laboratory from *Porphyridium cruentum* biomass, using a procedure analogous to that employed for phycocyanin isolation from *Spirulina maxima* (Section 4.2.1). For this study, phycoerythrin was typically excited with a 561 nm laser and its emission was detected at the 575–590 nm range. Microstructure transverse and surface views of the hydrogel were acquired using an EVO 50XVP confocal laser scanning microscope (Carl Zeiss, CZ Miscoscopy GmbHm, Jena, Germany). Ar/K and He/Ne dual-channel laser mode were used.

#### 4.2.5. Antioxidant Assessment by DPPH Colorimetric Assay

DPPH assay was used to determine the antioxidant ability of the antioxidants, namely, PC and CCM/BSA, when free in aqueous solution and when encapsulated into the hydrogel. The DPPH concentration was 50 mM in ethanol and the antioxidant’s concentration was 1 mg/mL in water and in the hydrogel, respectively. The absorbance of the DPPH solution without the presence of any antioxidant was used as a control. The reaction between DPPH and the free or encapsulated antioxidant leads to the reduction of the absorbance of the DPPH, thus giving information on the reaction yield and allowing the calculation of the antioxidant activity. The reaction time was 15 min and the absorbance was measured at 520 nm.

To study the antioxidant activity of the antioxidants incorporated into the hydrogels, a DPPH assay was performed on freeze-dried hydrogels. The freeze-dried hydrogel was inserted into a syringe with a 0.2 µm filter on the tip and moistened with DPPH solution. Then, 15 min later, the reaction solution was ejected from the syringe by the pressure of the plunger and was measured photometrically. The results were obtained as an average of three different measurements.

#### 4.2.6. Cell Culture and Cell Proliferation Assay

The human melanoma cell line WM164 (BRAFV600E, p53Y220C; Wistar Institute Melanoma Research Centre, https://wistar.org/) was generously provided by Dr. G. Skretas (National Hellenic Research Foundation, Athens, Greece). The cells were grown in Dulbecco’s Modified Eagle’s Medium (DMEM) containing 4.5 g/L glucose, L-glutamine, and pyruvate, supplemented with 10% FBS and 1% penicillin/streptomycin (Gibco-Life Technologies, Grand Island, NY, USA), at 37 °C in a humidified incubator with 5% CO_2_, as previously described [39].

The possible inhibition of cell proliferation due to the hydrogel’s presence was assessed for 48 and 72 h by MTT (3-[4,5-dimethylthiazol-2-yl]-2,5 diphenyl tetrazolium bromide) assay (M5655; Sigma-Aldrich) according to the manufacturer’s standard protocol. The assay is based on the conversion of MTT into formazan crystals by living cells, which determines mitochondrial activity. A 10 µL (10.4 mg) aliquot of the tested hydrogel was spread at the bottom of each well and was crosslinked by adding 1 µL of genipin solution (3 mg/mL) [40]. The mixture was incubated at 25 °C for 1 h to complete the crosslinking process. Subsequently, the samples were sterilized under UV light for 1 h and then incubated overnight at 37 °C under 5% CO₂ with 100 µL of culture medium (DMEM). The following day, the culture medium was replaced, and the cells were seeded into 96-well plates at a density of 1.2 × 10^4^ cells per well in 100 µL of fresh culture medium. This point was designated as time zero of the assay. The cells were incubated for 24 h at 37 °C under 5% CO₂. WM164 cells grow attached to the bottom of the well (adherent cells), therefore, the culture medium was refreshed at 24 h and incubation continued for another 24 h or 48 h (48 h or 72 h in total). Following this procedure, the empty or loaded hydrogel was used as a base on which cell cultures were settled to grow [concentrations: hydrogel, 10.4 μg/μL; PC, 0.4 μg/μL; CCM/BSA, 0.002 μg/μL]. Through this procedure, the developed hydrogels [empty hydrogel (blank), PC-loaded hydrogel, CCM/BSA-loaded hydrogel and hydrogel loaded with both, PC and CCM/BSA] were used to mimic natural extracellular matrix, an approach often followed in cell cultures to enhance cell proliferation [33].

MTT stock solution (5 mg/mL) was added to each culture being incubated for 48 or 72 h after crosslinking to equal one tenth of the original culture volume and further incubated for 3 h. At the end of the incubation, the solution was removed, and the insoluble formazan crystals were dissolved in 1:1 isopropanol/DMSO solution. The generated amount of blue formazan, reflecting viable cells, was measured at 470 nm (690 nm for background) using a Safire II TECAN microplate reader (Grödig, Austria). Cell viability was calculated using the following equation:

Cell viability (%) = OD of treated cells/OD of control × 100

where OD is the optical density.

All assays were carried out in triplicate. Statistical analysis was performed by One-Way ANOVA with Bonferroni correction for multiple testing.

## 5. Conclusions

Overall, the findings of the present study establish an alternative way to simultaneously encapsulate hydrophilic and hydrophobic biomolecules, maintaining their functionality. Chitosan–HPMC hydrogels were proven to be thermally stable and biocompatible materials with antioxidant properties. This study demonstrates the effectiveness of the phycocyanin purification process and highlights the potential of these hydrogels for biomedical applications, including food applications, drug delivery and tissue engineering. The results suggest that encapsulation can influence antioxidant activity and that the synergistic effects of chitosan along with the antioxidants may enhance bioactivity. These insights contribute to the ongoing development of functional biomaterials and provide a foundation for further optimization for food, pharmaceutical, and biomedical applications.

## Figures and Tables

**Table 1:** Composition of tested hydrogels. The weight of polymers is expressed as % *w*/*w* in the hydrogel.

Hydrogel	Polymer Weight % (*w*/*w*)	
	Chitosan	HPMC
A1	3.2	-
A2	2.4	-
A3	2.0	-
B1	-	16.7
B2	-	9.1
B3	-	6.3
C1	1.2	2.1
C2	1.0	1.0
C3	0.8	0.8
C4	1.7	1.7

**Table 2:** Antioxidant activity of PC, CCM/BSA, and their combination, as expressed by percentage of DPPH reduction after 15 min, in aqueous solution and when incorporated in hydrogels (system C4 in Table 1).

	% Reduction of DPPH	
	In Solution	In Hydrogel
Empty hydrogel	-	56 ± 0.01
PC	44 ± 0.04	54 ± 0.07
CCM/BSA	48 ± 0.07	71 ± 0.09
PC and CCM/BSA	58 ± 0.01	70 ± 0.02

## Data Availability

All research data are available upon request.

## Supplementary Materials

The following supporting information can be downloaded at https://www.mdpi.com/article/10.3390/ijms26083805/s1.

## Abbreviations

The following abbreviations are used in this manuscript:

HPMC	(Hydroxypropyl)methyl cellulose
PC	Phycocyanin
CCM	Curcumin
BSA	Bovine serum albumin
AS	Ammonium sulfate
PB	Phosphate buffer
DPPH	2,2-diphenyl-1-picryl-hydrazyl-hydrate
SEM	Scanning electron microscopy
CLSM	Confocal scanning electron microscopy
DMEM	Dulbecco’s Modified Eagle’s Medium
MTT	3-[4,5-dimethylthiazol-2-yl]-2,5 diphenyl tetrazolium bromide
