# Supplementary material for: Co-Encapsulation of Phycocyanin and Albumin-Bound Curcumin in Biopolymeric Hydrogels
Source: Int J Mol Sci. 2025 Apr 17;26(8):3805. doi: 10.3390/ijms26083805 (PMC12028146; doi:10.3390/ijms26083805)
Supplement: Supplementary file 1 [file ijms-26-03805-s001.zip › ijms-3544706-supplementary.pdf]

Supplementary Materials

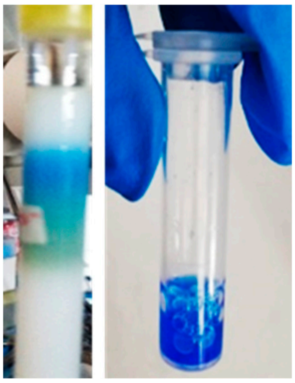

Figure S1. Left: DEAE Sepharose fast flow running sample of phycocyanin extract. Right: Purified PC after the chromatography purification.

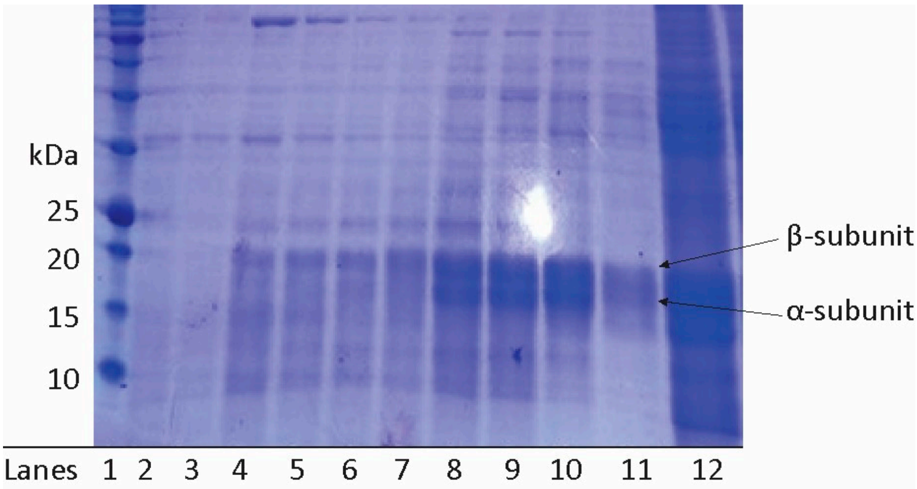

Figure S2. 1: Ladder; Samples 2-5: fractions by 100 mM PB; Samples 6-9: fractions occurring using 150 mM PB; Sample 10: fraction obtained by 200 mM PB; Sample 11: fraction obtained by 250 mM PB; Sample 12: sample retrieved with 1M NaCl during column cleaning process.

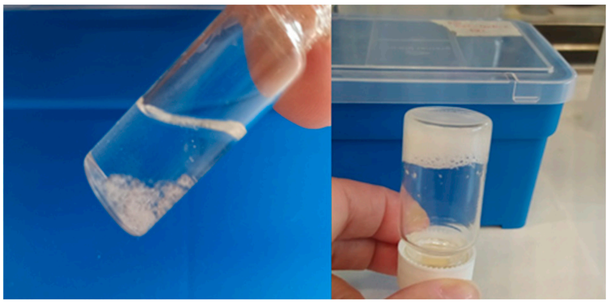

Figure S3. Hydrogel stability via Vial-tilt test, for System C1 of Table 1. Left: hydrogel in aqueous solvent; Right: inverted vial to demonstrate hydrogel stability.
